# Factors associated with institutional delivery in Ethiopia: a cross sectional study

**DOI:** 10.1186/s12913-020-05096-7

**Published:** 2020-03-31

**Authors:** Asmamaw Ketemaw, Minale Tareke, Endalkachew Dellie, Getachew Sitotaw, Yonas Deressa, Getasew Tadesse, Desta Debalkie, Mesafinet Ewunetu, Yibeltal Alemu, Daniel Debebe

**Affiliations:** 1 School of Public Health, College of Medicine and Health Science, Bahir Dar University, Bahir Dar, Ethiopia; 2 School of Medicine, College of Medicine and Health Science, Bahir Dar University, Bahir Dar, Ethiopia; 3 Institute of Public Health, College of Medicine and Health Science, University of Gondar, Gondar, Ethiopia; 4 Bahir Dar Institute of Technology, Bahir Dar University, Bahir Dar, Ethiopia

**Keywords:** Determinants, Institutional, Delivery, Ethiopia, Survey

## Abstract

**Background:**

In spite of the promotion of institutional delivery in Ethiopia, home delivery is still common primarily in hard-to-reach areas. Institutional delivery supported to achieve the goal of reducing maternal and neonatal mortality in Ethiopia. The objective of this study is to assess the determinants of institutional delivery in Ethiopia.

**Methods:**

Cross sectional survey was conducted in 11 administrative regions of Ethiopia. The Ethiopian demographic and health survey data collection took place from January 18, 2016, to June 27, 2016. The study subjects were 11,023 women (15–49 years old) who gave birth in the preceding 5 years before 2016 Ethiopian demographic health survey. This representative data was downloaded from Demographic Health Survey after getting permission. The Primary outcome variable was institutional delivery. The data was transferred and analyzed with SPSS Version 20 statistical software package.

**Results:**

Of 11,023 mothers, 2892 (26.2%) delivered at a health facility and 8131 (73.8%) at home. Women with secondary education were 4.36 times more likely to have an institutional delivery (OR: 4.36; 95% CI: 3.12–6.09). Institutional delivery was higher among women who were resided in urban areas by three fold (OR: 3.26; 95% CI: 2.19–4.35). Women who visited ANC (Antenatal care) were about two times more likely to choose institutional delivery (OR: 1.81; 95% CI: 1.58–2.07). Respondents who watch television at least once a week was two times more likely to experience institutional delivery than those who did not watch at all (0R: 1.90; 95% CI: 1.35–2.66). The wealthiest women were 2.61 times more likely to deliver in an institution compared with the women in the poorest category (OR: 2.61; 95% CI: 1.95–3.50).

**Conclusion:**

Women having higher educational level, being richest, residing in urban area, visiting antenatal care at least once, and frequent exposure to mass media were factors associated with institutional delivery. Improving access to education and health promotion about obstetrics and delivery through mass media will increase the uptake of institutional delivery.

## Background

Worldwide, about 140 million women give birth every year and about 890 women die from causes related to pregnancy and childbirth every day. Most of these problems were preventable. Developing countries took 99% of all maternal deaths in the world [1]. Of which, 66% were in Sub Saharan Africa. The major direct cause of maternal morbidity and mortality include hemorrhage, infection, high blood pressure, unsafe abortion, and obstructed labor [1–3].

The increment of institutional delivery is important for reducing maternal and neonatal mortality [4]. High attention and effort are being given to reach target populations with specific programmatic strategies. This is more valuable, especially in countries that are not achieved the Million Development Goals (MDGs) [5]. In 2015, approximately 125 million women gave birth; of which 303,000 died, leaving many children without a mother. Pregnancy-related deaths that occurred during pregnancy or childbirth, or within 42 days after the birth or termination of a pregnancy is estimated to be 25% in Ethiopia [4, 6]. One of the contributing factor can be access to health facilities in rural areas is more difficult than in urban areas.

Health facilities in rural settings are distant, inaccessible, with limited resources and scares health professionals [4]. Although institutional delivery has been promoted in Ethiopia, home delivery is still common, primarily in hard-to-reach areas [2–4]. Women’s delivery with the assistance of a skilled birth attendant is one of the indicators in meeting the fifth MDG that is improving maternal health. In most countries where health professionals attend more than 80% of deliveries, Maternal Mortality Rate (MMR) is below 200 per 100,000 live births [7]. Even though there is a sharp decline in home delivery in Ethiopia, it was higher than some African countries, like Kenya [4, 8]. Maternal health programs should be designed to encourage young women to receive adequate Antenatal Care (ANC), at least four times. ANC visits at least four times had a strong and positive association with the use of institutional delivery [9]. Lower income to utilize maternal health services and the lack of knowledge on delivery care from a trained provider remain substantial barriers for institutional delivery [3, 9–11]. Physical access to health facilities and/or lack of transport were also hinder women’s to deliver in a health facility [8].

Generally, Institutional delivery was still lower in Ethiopia that was below 30% in most studies, although there was a progress in the past two decades [4, 6]. Therefore, this study will assess the determinants of institutional delivery in Ethiopia from the Ethiopian Demographic Health Survey datasets.

## Methods

### Study setting and period

The population of Ethiopia is diverse encompassing 80 different ethnic groups. According to the 2017 estimate, the population of Ethiopia was about 107,406,158. Ethiopian population is equivalent to 1.41% of the total world population, which ranks number 12 from the world. About 20% of Ethiopian population were resided in urban areas [12, 13]. The Ethiopian demographic and health survey data collection took place from January 18, 2016, to June 27, 2016. The data collectors were health professionals recruited from different health facilities throughout the country. Women in reproductive age (15–49) who resides permanently in the selected households or stayed the night before the survey in the household, were entitled to be part of the study subjects.

### Study design and sampling

Cross sectional survey was conducted in 11 administrative regions of Ethiopia. The sample was stratified and selected in two stages where each region stratified into urban and rural areas. Samples of enumeration areas (EAs) selected independently in each stratum in two stages. Based on the 2007 population and housing census, in the first stage, 645 EAs of which 202 from urban areas and 443 from rural areas were selected. Household lists helped as a sampling frame for second stage selection of households for the study. Up to 300 households listed in one-enumeration areas. To reduce the task of household listing, each large enumeration areas selected for the survey was segmented. Then household listing was done only in the selected segments. In the second stage of selection, a fixed number of 28 households in each cluster selected with an equal probability of systematic selection from the newly created household listing.

### Study participants

The sampling frame of the 2016 Ethiopian demographic health survey was from Ethiopia Population and Housing Census (PHC), conducted by the Ethiopia Central Statistical Agency in 2007 [4]. About 16,583 entitled women identified for individual interviews. Then Interview completed with 15,683 respondents resulting in the response rate of 95% [4]. To assess determinants of institutional delivery, mothers who gave baby within the preceding 5 years extracted from EDHS dataset. Therefore, 11,023 women included in this study.

### Sampling weight

Since there was a non-proportional allocation of the sample to different regions and their urban and rural areas and the possible differences in response rates, a sampling weight used to ensure the actual representative of the survey results at both the national and domain levels. The sample was taken in a two-stage stratified cluster sample. Therefore, the sampling weights are based on sampling probabilities separately for each sampling stage and each cluster [4].

### Study variables

The outcome variable of the study is institutional delivery. Since the study wants to answer the question “what are the determinants of institutional deliveries?” women who deliver at health institutions at least once will be coded as institutional delivery (Yes = 1). The independent variables where socio demographic characteristics (maternal age, marital status place of resident, maternal educational, husband education, wealth index and watching television), pregnancy and health service related factors (Number of Births, antenatal care, told about danger sign during ANC, and health insurance).

### Data quality

Data files transferred via internet file streaming system (IFSS) to the CSA central office during data collection. The data processing operation included secondary editing, which required resolution of computer-identified inconsistencies and coding of open-ended questions. Data editing was accomplished using CSPro software. During the duration of fieldwork, tables generated to check various data quality parameters [4] and specific feedback was given to the teams to improve performance.

### Statistical analysis

Bivariate logistic regression employed, with the outcome variable of intuitional delivery. First, univariable analyses performed with each of the demographic indicators and other independent variables with the outcome variable. Variables significant at *p*-value ≤0.2 were included in the multivariable logistic regression models. Variables that did not have a significant regression coefficient removed from the model. Variables that were not significant at the univariate analysis added back to the model and their significance assessed in the presence of other significant variables. Subsequently, the goodness of fit of our final model tested using the Hosmer-Lemeshow test. Data management procedures and statistical analysis done with SPSS software version 20.

## Results

### Background characteristics of study participants

A total of 11,023 mothers who gave birth within the preceding five years were included in the study. The mean age of the participants were 29.5 (SD ± 6.6). The age range was between 15 to 45. Most of the women resided in rural areas, 9807 (89%). Majority of them were married (93.8%). Christianity and Muslim were the prominent religions among the respondents, which were 55.3 and 41.4% respectively. Above half of the women and their husbands had no formal education, 61.1 and 51.5% respectively.

Of 11,023 mothers, 2892 (26.2%) delivered at a health facility (public or private sector health facility) and 8131 (73.8%) at their or others home. About half of the respondents were on their 4th and beyond births which was 5605 (50.9%) as described in Table 1 below. 4772 (62,9%) women visited ANC at least once during their pregnancy. In addition, from all women who visited ANC at least once, about 2145 (45%) women told about the danger signs of pregnancy during their follow-up. About half of the women (46.8%) were in the poorest and poorer category, from the five-wealth index category. A very small number of women, 390 (3.5%) covered by health insurance in the study.

**Table 1:** Background characteristics of study participants, Ethiopian DHS 2016

Variables	Frequency	Percentage
Place of delivery grouped		
Health Institution	2892	26.2
Home	8131	73.8
Maternal Age		
15–24	2446	22.2
25–34	5843	53.0
35–49	2734	24.8
Type of place of residence		
Urban	1216	11.0
Rural	9807	89.0
Current marital status		
No	684	6.2
Yes	10,339	93.8
Maternal Education		
No education	7284	61.1
Primary	2951	26.8
Secondary	514	4.7
Higher	274	2.5
Husband Educational Level		
No education	5077	48.5
Primary	4116	39.3
Secondary	798	7.6
Higher	471	4.5
Wealth index		
Poorest	2636	23.9
Poorer	2520	22.9
Middle	2280	20.7
Richer	1999	18.1
Richest	1588	14.4
Religion		
Orthodox	3772	34.2
Protestant	2329	21.1
Muslim	4561	41.4
Others	361	3.3
Number of Births		
First delivery	2058	18.7
2nd and 3rd delivery	3359	30.5
4th delivery and above	5605	50.9
The frequency of watching television		
Not at all	9019	81.8
Less than once a week	1089	9.9
At least once a week	915	8.3
Covered by health insurance		
No	10,633	96.5
Yes	390	3.5
Told about danger signs during ANC		
No	2627	55.0
Yes	2145	45.0
ANC Visit		
No	2818	37.1
Yes	4772	62.9
Total	**11,023**	**100.0**

### Determinants of institutional delivery

Variables that appeared to be associated with institutional delivery included; age of the women, women’s level of education, religion, place of residence, wealth, ANC visits and frequency of watching television. The multivariate analysis on Table 2 showed that women’s age had a significant effect on the use of institutional delivery among young women. After controlling for the other socio-demographic variables, women between 15 and 24 years of age had a significantly higher number of institutional deliveries as compared with the older women. Women who attended secondary education were 4.36 times more likely to have an institutional delivery as compared with those who had no formal education (OR: 4.36; 95% CI: 3.12–6.09).

**Table 2:** Multivariate analysis for the determinants of institutional delivery in Ethiopia, EDHS 2016

Variables	COR (95% CI)	AOR (95% CI)
Age Group		
15–24	1.00	1.00
25–34	0.66 (0.59–0.73)	0.679 (0.56–0.79)
35–49	0.50 (0.44–0.56)	0.68 (0.55–0.83)
Currently Married		
No	1.00	1.00
Yes	1.47 (1.25–1.74)	0.81 (0.63–1.06)
Religion		
Orthodox	1.00	1.00
Protestant	0.55 (0.49–0.62)	0.55 (0.46–0.65)***
Muslims	0.44 (0.40–0.48)	0.73 (0.62–0.85)***
Others	0.18 (0.13–0.26)	0.31 (0.18–0.52)***
Residence		
Rural	1.00	1.00
Urban	7.06 (5.44–8.61)	3.26 (2.19–4.35)***
Highest Educational Level		
No education	1.00	1.00
Primary	3.07 (2.79–3.39)	1.57 (1.35–1.83)***
Secondary	18.10 (14.58–22.46)	4.36 (3.12–6.09)***
Higher	56.70 (36.94–87.01)	4.49 (2.50–8.06)***
Wealth Index		
Poorest	1.00	1.00
Poorer	1.93 (1.65–2.27)	1.50 (1.20–1.87)
Middle	2.42 (2.06–2.83)	1.54 (1.24–1.92)
Richer	3.17 (2.71–3.71)	1.61 (1.28–2.01)
Richest	18.47 (15.69–21.74)	2.61 (1.95–3.50)
Covered by Health Insurance		
No	1.00	1.00
Yes	2.35 (1.91–2.88)	1.30 (0.96–1.76)
Antenatal Care Visit		
No	1.00	1.00
Yes	5.20 (4.52–5.99)	1.81 (1.58–2.07)***
Told about danger sign during visit		
No	1.00	1.00
Yes	2.01 (1.79–2.26)	1.41 (1.23–1.62)***
The frequency of watching television		
Not at all	1.00	1.00
Less than once a week	2.45 (2.14–2.79)	1.32 (1.07–1.63)
At least once a week	16.02 (13.53–18.97)	1.90 (1.35–2.66)**
Number of Births		
First delivery	1.00	1.00
2nd and 3rd delivery	2.287 (2.040–2.564)	1.678 (1.426–1.975)
4th delivery and above	4.748 (4.248–5.308)	2.165 (1.784–2.626)

**p < 0.01, ***p < 0.001

Regarding their place of residence, the probability of giving birth in an institution was higher among women who resided in urban areas by three fold than their counterparts were, (OR: 3.26; 95% CI: 2.19–4.35). Women who had received at least one ANC visits were about two times more likely to deliver at health institution than women who did not receive ANC at all, or got birth without ANC follow-up (OR: 1.81; 95% CI: 1.58–2.07). The probability of giving birth at health institution is higher for women who told about danger signs of pregnancy by 41% (OR: 1.41; 95% CI: 1.23–1.62), during follow-up by their care providers. Exposure to mass media had also a significant association with institutional delivery. Women who watch television at least once a week was two times more likely to deliver their child at health institution than women who did not watch at all (0R: 1.90; 95% CI: 1.35–2.66). The socioeconomic status of a woman determines the institutional delivery. Wealthiest women were 2.61 times more likely to deliver in the health facility as compared with women in the poorest wealth index level (OR: 2.61; 95% CI: 1.95–3.50). Women in their forth delivery and beyond had higher probability to get birth in the health institution with two folds as compared to those women in their first delivery (OR: 2.16: 95% CI: 1.784–2.626).

## Discussion

Though institutional delivery is vital for reducing maternal and neonatal mortality, the figure shows, as it was lower in Ethiopia, 26% [4]. Therefore, this study aims to assess the determinants of institutional delivery in Ethiopia. In this study; younger women, being orthodox christian religion follower, residing in urban area, maternal higher education, being richest, having antenatal care visit, telling danger signs of pregnancy for the women and watching television had a significant association with institutional delivery.

Younger women between the ages of 15–24 had better institutional delivery than older women in contrast to studies done in Kenya, Uganda, Guinea and Malawi [3, 8, 11, 14], where age of the women doesn’t have significant association with health facility delivery. Women who had formal education delivered their babies in health institutions than those without education. This finding was consistent with many studies done in African and Asian countries with similar socio-economic status [3, 8–11, 14]. Since education boosts the need for information and knowledge, the increase in the educational level of the women influenced women to visit health facilities. Economic status of the women was another determinant factor. This finding was supported by studies in some African and Asian low and middle income countries; Kenya, Malawi, Guinea, Nepal and Pakistan [8–11, 14], In which wealthiest women were more likely to deliver in the health facility than the poor. The reason may be lack of money to cover transportation and health service costs that leads them to give birth at home with traditional birth attendants. Even though health expenditures were covered for women who had community health insurance, most women in this study were not a member of this scheme (96.5%), as indicated in Table 1.

The provision of antenatal care helped mothers to give birth at health facilities; however, antenatal care visit in this study was lower than some African countries. A study in Kenya and Guinea shows that 92.6 and 81.7% mothers visited antenatal care at least once during their pregnancy, respectively [8, 14]. Women who had ANC visit were about two times more likely to choose institutional delivery than women who did not have ANC at all. This finding was similar with a study done in Kenya were young women who had more than four antenatal care (ANC) visits were three times more likely to deliver in a health institution compared with women who had no antenatal care visit [8]. Counseling during ANC and telling of women about danger signs of pregnancy via care providers may be the reason for increased institutional delivery. Telling about danger signs of pregnancy for pregnant women positively influences institutional delivery. Women who told about danger signs of pregnancy during ANC were 41% more likely to use health facilities for delivery, which is consistent with the study done in Uganda and Tanzania [2, 3]. Knowing the danger signs of pregnancy will increase women’s attitude towards pregnancy care and their health-seeking behavior that leads them to deliver at the health institution.

Mass media exposure influenced institutional delivery as shown in the study. Women who watch television at least once a week were two times more likely to undergo institutional delivery. A similar study at Pakistan also describes, exposure to mass media were important drivers of institutional delivery [10]. Number of births was also associated with institutional delivery. As number of births increased, women tend to deliver at health institutions. This may be due to previous pregnancy and birth experience may influence women attitude towards institutional delivery. As a limitation, since the study was done using secondary data collected for Ethiopian Demographic Health Survey, it was difficult to research the variable of interest. Also reverse causality due to the cross-sectional study design and confounding bias may be the potential limitation for the study. Since institutional delivery was highly influenced by antenatal care visits, it was recommended to do further study on factors that enhance antenatal care visits.

## Conclusion

Women having higher educational level, being richest, residing in urban area, visiting antenatal care at least once, and frequent exposure to mass media were important determinant factors for institutional delivery. Improving access to education and health promotion about obstetrics and delivery through mass media will increase the uptake of institutional delivery.

## Data Availability

The datasets analyzed during the current study are available in the DHS program repository, available at https://dhsprogram.com/data/dataset_admin/ up on permission.

## Abbreviations

EDHS: Ethiopian Demographic Health Survey
ANC: Antenatal Care
DHS: Demographic Health Survey
CSA: Central Statistics Agency
COR: Crude Odds Ratio
AOR: Adjusted Odds Ratio
